# Association between visit-to-visit glycated hemoglobin A1c variability and recurrent falls over 12 months in older adults with type 2 diabetes mellitus

**DOI:** 10.3389/fendo.2026.1971401

**Published:** 2026-10-22

**Authors:** Wei Hu, Anjing Shao

**Affiliations:** 1 Department of Gastroenterology, Chongqing Jiulongpo District People’s Hospital, Chongqing, China; 2 Department of Endocrinology, Chongqing Wanzhou District Shanghai Hospital, Chongqing, China

**Keywords:** glycated hemoglobin A1c, glycemic variability, older adults, recurrent falls, type 2 diabetes

## Abstract

**Background:**

Visit-to-visit glycated hemoglobin A1c (HbA1c) variability may capture glycemic instability beyond mean HbA1c, but its association with recurrent falls in older adults remains uncertain.

**Methods:**

This single-center retrospective cohort study included 286 adults aged ≥65 years with type 2 diabetes who received outpatient follow-up at Chongqing Jiulongpo District People’s Hospital and had at least four HbA1c measurements during a 365-day exposure window. Recurrent falls were defined as at least two independent falls during the subsequent 365 days. Modified Poisson regression with robust variance estimated risk ratios (RRs) for the HbA1c coefficient of variation (HbA1c-CV); negative binomial regression assessed fall counts.

**Results:**

Recurrent falls occurred in 77 participants (26.9%). The median HbA1c-CV was 7.0%, and its between-patient sample standard deviation (SD) was 3.1 percentage points. Each 1-SD increase in HbA1c-CV was associated with recurrent falls after adjustment (RR, 1.34; 95% confidence interval [CI], 1.16–1.55; *P* < 0.001). Compared with the lowest tertile, the highest tertile had an adjusted RR of 2.03 (95% CI, 1.19–3.47; *P* = 0.010). Restricted cubic spline analysis showed no evidence of nonlinearity (*P* = 0.294). Higher HbA1c-CV was also associated with a higher fall incidence rate (adjusted incidence rate ratio per 1-SD increase, 1.37; 95% CI, 1.18–1.58; *P* < 0.001).

**Conclusion:**

Greater visit-to-visit HbA1c variability was associated with recurrent falls and a higher fall burden in older adults with type 2 diabetes. HbA1c-CV may complement multidimensional fall-risk assessment.

## 1 Introduction

Population aging has shifted the management of type 2 diabetes in older adults from a sole focus on glucose-lowering targets toward comprehensive care that addresses functional status, hypoglycemia prevention, and long-term complications (1). Falls are common adverse clinical events in older adults and may recur; risk assessment therefore needs to consider previous falls, gait and balance, medications, and environmental factors (2). Older adults with diabetes have a higher risk of falls than those without diabetes, and factors such as age, diabetes duration, and insulin therapy may further increase this risk (3). Severe hypoglycemia can transiently impair consciousness, responsiveness, and postural control and is significantly associated with falls (4). Reduced plantar sensation, impaired proprioception, and decreased gait stability due to diabetic peripheral neuropathy may also compromise the ability to compensate for environmental perturbations (5).

Glycated hemoglobin A1c (HbA1c) reflects longer-term average glycemic exposure, but a single HbA1c value or mean HbA1c does not characterize the stability of glycemic control between visits. Greater visit-to-visit HbA1c variability is independently associated with several adverse diabetes-related outcomes (6). Even when mean HbA1c is within the target range, long-term fluctuations may provide prognostic information distinct from that provided by the mean level (7). Less time with HbA1c within individualized target ranges is associated with worse cognitive outcomes, suggesting that long-term glycemic stability may affect functional reserve in older patients (8). Greater HbA1c variability is also associated with limitations in mobility and activities of daily living (9), while glycemic control in older adults with diabetes is clinically associated with fall risk (10).

Direct evidence linking visit-to-visit HbA1c variability to recurrent falls remains limited. Mean HbA1c, previous falls, severe hypoglycemia, functional status, and medication burden may jointly influence this association. This study evaluated the association between visit-to-visit HbA1c variability and recurrent falls over the subsequent 12 months in older adults with type 2 diabetes, and assessed the robustness of the findings using alternative exposure measures, complete-case analysis, and penalized regression.

## 2 Materials and methods

### 2.1 Study population

This single-center retrospective cohort study was conducted in the Department of Gastroenterology at Chongqing Jiulongpo District People’s Hospital. The source population comprised consecutive older adults with type 2 diabetes receiving standardized diabetes management, identified from the hospital information system, outpatient records, and existing follow-up records. Patients with index dates between January 1, 2023, and December 31, 2024, were consecutively screened. For each patient, the index date and the preceding 364 days constituted the exposure assessment window, and days 1–365 after the index date constituted the outcome observation window. Accordingly, the required clinical records covered January 1, 2022, through December 31, 2025.

The inclusion criteria were: (1) a diagnosis of type 2 diabetes according to established diagnostic criteria and a disease duration of ≥1 year (11); (2) age ≥65 years at the index date and receipt of routine care appropriate for older adults with diabetes (12); (3) at least four routine outpatient HbA1c measurements during the exposure assessment window, with an interval of ≥8 weeks between consecutive measurements and a span of ≥9 months between the first and last measurements; (4) the ability to walk independently or with an assistive device; and (5) complete ascertainment of fall outcomes during the 365 days after the index date. The exclusion criteria were type 1 diabetes, gestational diabetes, or other specific types of diabetes; conditions affecting the interpretation of HbA1c results; long-term bed confinement, complete wheelchair dependence, or inability to walk; unavailable original HbA1c records; or fall outcomes that could not be reliably ascertained.

The study protocol was reviewed and approved by the Medical Ethics Committee of Chongqing Jiulongpo District People’s Hospital (approval number: 20260802). The study used anonymized historical clinical data, and the requirement for written informed consent was waived. The study was conducted in accordance with the Declaration of Helsinki.

### 2.2 HbA1c measurement and visit-to-visit variability

HbA1c was measured using ion-exchange high-performance liquid chromatography and reported in United States National Glycohemoglobin Standardization Program units (%). Testing and quality control followed recommendations for laboratory analysis in diabetes (13). Only quality-controlled results obtained during routine outpatient care were included. Measurements obtained on an *ad hoc* basis during acute hospitalizations were excluded from the calculations. When duplicate results were available within the same 8-week period, the first routine measurement was retained. All outlying values were checked against the original laboratory records and medical records.

Chronologically ordered measurements were used to calculate each patient’s mean HbA1c, within-person standard deviation (SD), and coefficient of variation (CV), with CV (%) = (SD/mean) × 100%. The primary exposure was the HbA1c coefficient of variation (HbA1c-CV), analyzed per 1-SD increase and by tertile. For this exposure, 1 SD denotes the between-patient sample SD of HbA1c-CV, equivalent to 3.1 percentage points, rather than the within-person SD of HbA1c. Alternative exposure measures included the HbA1c standard deviation (HbA1c-SD), variability independent of the mean (VIM), and the residual SD after removal of the within-person linear time trend. The definitions of these measures were consistent with those used in recent research on long-term HbA1c variability (14).

### 2.3 Fall outcomes and observation endpoints

A fall was defined as an event in which an individual unexpectedly came to rest on the ground, floor, or another lower level. Events caused by intentional acts, traffic accidents, falls from a height, or major external forces were not classified as falls (15). The primary outcome was recurrent falls, defined as at least two independent falls within 365 days after the index date. A subsequent fall was considered a new event if the patient had returned to the pre-event level of activity before it occurred. Outpatient, emergency department, inpatient, and imaging records pertaining to the same fall were consolidated according to the date, location, injury site, and event description.

Secondary outcomes were the occurrence of any fall and the total number of falls during the observation period. Fall information was obtained from structured outpatient records, existing telephone follow-up records, emergency department and inpatient records, nursing records, injury or fracture diagnoses, and imaging data, and was not limited to events resulting in injury or requiring medical attention. Two investigators blinded to HbA1c variability independently adjudicated the events and their dates; disagreements were reviewed by a third investigator. Outcome status throughout the 365-day period was ascertained for all included patients.

### 2.4 Covariates

Covariates were obtained from records on the index date or the most recent eligible records before that date. They included age, sex, body mass index, diabetes duration, living alone, a history of falls during the exposure assessment window, a history of severe hypoglycemia, limitations in activities of daily living, use of a walking aid, cognitive impairment, visual impairment, diabetic peripheral neuropathy, cerebrovascular disease, orthostatic hypotension, and estimated glomerular filtration rate (eGFR). Diabetic peripheral neuropathy was identified using clinical symptoms, signs, and documented standardized foot sensory assessments (16). A limitation in activities of daily living was defined as requiring assistance with at least one of the following: bathing, dressing, toileting, transferring, feeding, or continence (17). Severe hypoglycemia was defined as a hypoglycemic event requiring assistance from another person (18).

Active prescriptions at the index date were recorded for insulin, sulfonylureas or glinides, sedative–hypnotics, antidepressants, antipsychotics, opioids, antihypertensives, and diuretics. Treatment carrying a high risk of hypoglycemia was defined as the use of insulin and/or sulfonylureas or glinides. The number of fall-risk-increasing medication classes was the sum of the classes currently used among sedative–hypnotics, antidepressants, antipsychotics, opioids, antihypertensives, and diuretics, consistent with the medication recommendations in the World Guidelines for Falls Prevention and Management (19). The total number of long-term medications and the number of changes to the glucose-lowering regimen during the exposure assessment window were also recorded.

### 2.5 Statistical analysis

Continuous variables are presented as mean ± SD or median [interquartile range (IQR)], according to their distribution, and were compared between groups using the independent-samples *t* test or the Mann–Whitney *U* test. Categorical variables are presented as counts and percentages and were compared using the *χ²* test or Fisher’s exact test. The 365-day risk of recurrent falls in each HbA1c-CV tertile was calculated as the number of patients with recurrent falls divided by the number of patients in that tertile. The 95% confidence intervals (CIs) were calculated using the Wilson method, and between-group comparisons were performed using Pearson’s *χ²* test.

The primary analysis used modified Poisson regression with a log link and robust sandwich variance to estimate risk ratios (RRs) and 95% CIs. The prespecified adjustment variables were age, mean HbA1c, the number of HbA1c measurements, the span between the first and last measurements, a history of falls during the exposure assessment window, a history of severe hypoglycemia, limitations in activities of daily living, and treatment carrying a high risk of hypoglycemia. Variables were not selected on the basis of univariable *P* values. Restricted cubic splines were fitted with three knots at the 10th, 50th, and 90th percentiles of HbA1c-CV, corresponding to 3.8%, 7.0%, and 11.3%, respectively, with 7.0% as the reference. Overall association and nonlinearity were assessed using pooled Wald tests.

Negative binomial regression was used to analyze the total number of falls within 365 days, with results reported as incidence rate ratios (IRRs) and 95% CIs. Overdispersion was evaluated using the dispersion parameter *α* of the negative binomial model, the Pearson dispersion statistic of the corresponding Poisson model, and a boundary-corrected likelihood ratio test. Sensitivity analyses included replacing HbA1c-CV with HbA1c-SD, VIM, or residual SD; restricting the analysis to patients with exactly four HbA1c measurements; excluding patients who had fallen during the exposure assessment window; complete-case analysis; and ridge-penalized modified Poisson regression with an expanded set of adjustment variables.

Missing covariate data were handled using multiple imputation by chained equations, generating 20 imputed datasets. The imputation model included all analysis variables and the primary outcome indicator. Predictive mean matching was used for continuous variables, and logistic regression was used for binary variables. Except for the complete-case analysis and the expanded ridge-penalized analysis, multivariable models were fitted to the imputed data, and regression coefficients and standard errors were pooled using Rubin’s rules. Variance inflation factors were calculated separately in each of the 20 imputed datasets, and the overall range was reported. All tests were two-sided, and *P* < 0.05 was considered statistically significant. Secondary and sensitivity analyses were exploratory, and their *P* values were not adjusted for multiplicity. Analyses were performed using R version 4.3.2.

## 3 Results

### 3.1 Patient selection and baseline characteristics

Records for 341 patients were identified. Fifty-five patients were excluded: 23 because the number or time span of HbA1c measurements did not meet the eligibility requirements, 12 because fall information could not be reliably verified, seven because of conditions affecting the interpretation of HbA1c results, eight because they could not walk independently or with an assistive device, and five because they had other types of diabetes. The final analysis included 286 patients, of whom 77 experienced recurrent falls and 209 did not during the observation period (**Figure 1**).

**Figure 1:**
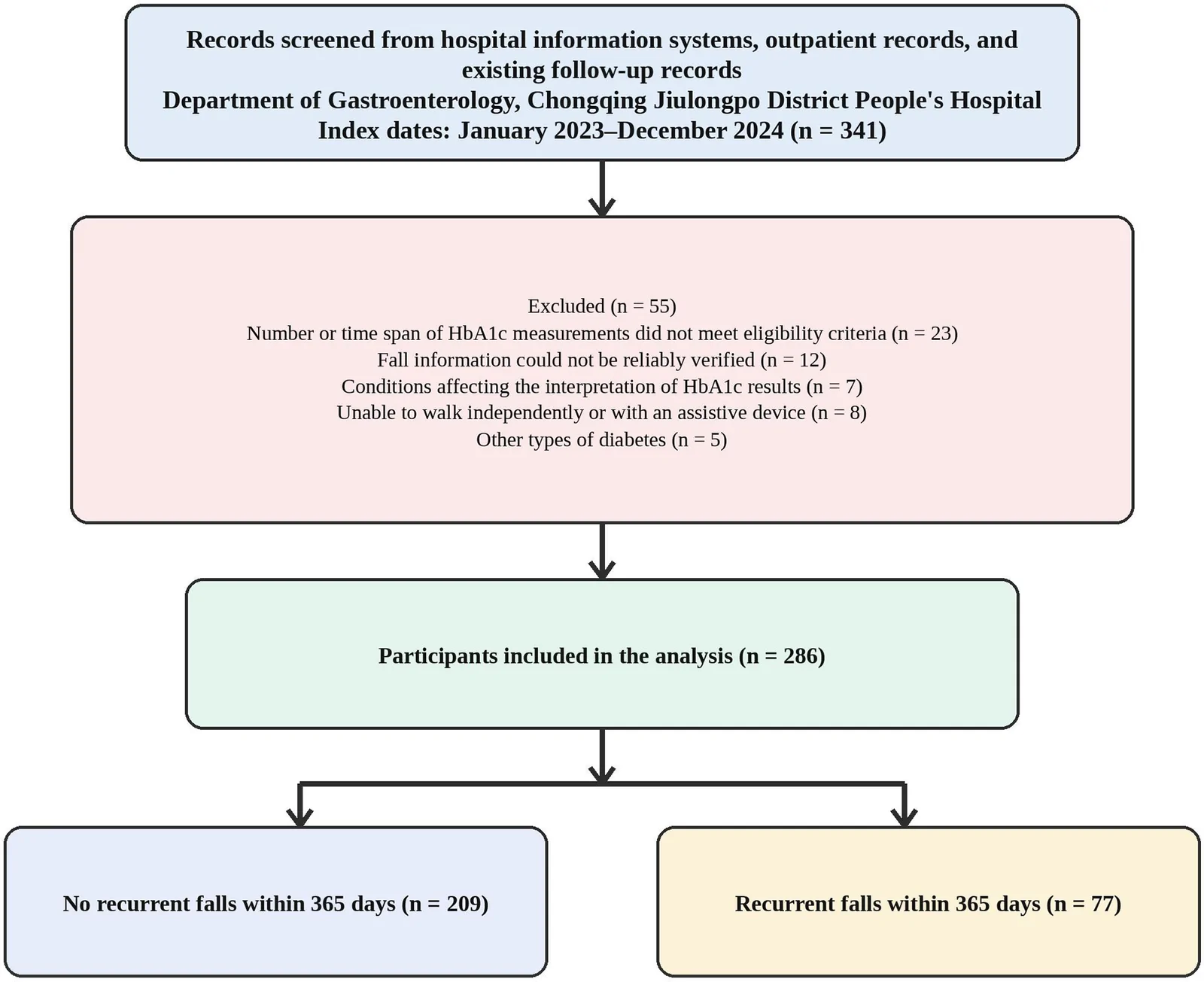
Participant selection flowchart. Records for 341 consecutive patients were screened, and 286 patients were included after 55 exclusions. During the 365-day observation period, 77 patients experienced recurrent falls and 209 did not. HbA1c, glycated hemoglobin A1c; *n*, number of patients.

Compared with patients without recurrent falls, those with recurrent falls were older, had a longer diabetes duration, and more frequently lived alone or had a history of falls during the exposure assessment window, severe hypoglycemia, limitations in activities of daily living, walking-aid use, cognitive impairment, visual impairment, diabetic peripheral neuropathy, cerebrovascular disease, or orthostatic hypotension. They also had lower eGFR (all *P* < 0.05). The proportion of women and body mass index did not differ significantly between the groups (both *P* > 0.05) (**Table 1**).

**Table 1:** Baseline characteristics of patients with and without recurrent falls.

Variable	Overall (*N* = 286)	No recurrent falls (*n* = 209)	Recurrent falls (*n* = 77)	Statistic	*P* value
Age, years	73.6 ± 5.9	72.8 ± 5.6	75.6 ± 6.2	*t* = 3.642	<0.001
Female, *n* (%)	160 (55.9)	110 (52.6)	50 (64.9)	*χ²* = 3.456	0.063
Body mass index, kg/m² (*n* = 279; 204/75)	24.8 ± 3.2	24.9 ± 3.2	24.4 ± 3.2	*t* = −1.157	0.248
Diabetes duration, years	12 [8–18]	11 [7–16]	15 [10–21]	*Z* = 3.870	<0.001
Living alone, *n* (%)	47 (16.4)	27 (12.9)	20 (26.0)	*χ²* = 6.984	0.008
Falls during the exposure assessment window, *n* (%)	88 (30.8)	45 (21.5)	43 (55.8)	*χ²* = 31.101	<0.001
History of severe hypoglycemia, *n* (%)	30 (10.5)	14 (6.7)	16 (20.8)	*χ²* = 11.882	<0.001
Limitations in activities of daily living, *n*/*N* (%)	59/282 (20.9)	30/206 (14.6)	29/76 (38.2)	*χ²* = 18.681	<0.001
Walking-aid use, *n* (%)	53 (18.5)	29 (13.9)	24 (31.2)	*χ²* = 11.146	0.001
Cognitive impairment, *n* (%)	31 (10.8)	16 (7.7)	15 (19.5)	*χ²* = 8.142	0.004
Visual impairment, *n* (%)	74 (25.9)	43 (20.6)	31 (40.3)	*χ²* = 11.369	0.001
Diabetic peripheral neuropathy, *n* (%)	110 (38.5)	66 (31.6)	44 (57.1)	*χ²* = 15.536	<0.001
Cerebrovascular disease, *n* (%)	61 (21.3)	37 (17.7)	24 (31.2)	*χ²* = 6.080	0.014
Orthostatic hypotension, *n* (%)	41 (14.3)	22 (10.5)	19 (24.7)	*χ²* = 9.173	0.002
Estimated glomerular filtration rate (eGFR), mL/min/1.73 m² (*n* = 280; 204/76)	71.3 ± 17.9	73.4 ± 17.2	65.8 ± 18.3	*t* = −3.231	0.001

*N*, total sample size; *n*, number of patients in a group; *n*/*N*, number of patients with the event or characteristic/number of patients with available data; SD, standard deviation; IQR, interquartile range; *t*, independent-samples *t*-test statistic; *χ²*, Pearson chi-square statistic; *Z*, standardized statistic for the Mann–Whitney *U* test; eGFR, estimated glomerular filtration rate. Continuous variables are presented as mean ± SD or median [IQR]. The values 204/75 and 204/76 in parentheses indicate the numbers of patients with available data in the groups without/with recurrent falls, respectively.

### 3.2 HbA1c variability and medication characteristics

Mean HbA1c, the number of HbA1c measurements, HbA1c-SD, HbA1c-CV, VIM, and residual SD were higher in patients with recurrent falls than in those without recurrent falls (all *P* < 0.05). The proportion of patients with exactly four HbA1c measurements and the span between the first and last measurements did not differ significantly between the groups (both *P* > 0.05).

Patients with recurrent falls more frequently used insulin, sulfonylureas or glinides, treatment carrying a high risk of hypoglycemia, sedative–hypnotics, and diuretics. They also had more long-term medications, more fall-risk-increasing medication classes, and more changes to their glucose-lowering regimens (all *P* < 0.05). The proportions using antidepressants, antipsychotics, opioids, and antihypertensives did not differ significantly between the groups (all *P* > 0.05) (**Table 2**).

**Table 2:** Glycated hemoglobin A1c (HbA1c) variability and medication characteristics.

Variable	Overall (*N* = 286)	No recurrent falls (*n* = 209)	Recurrent falls (*n* = 77)	Statistic/test	*P* value
Mean HbA1c, %	7.58 ± 0.80	7.52 ± 0.77	7.76 ± 0.84	*t* = 2.281	0.023
Number of HbA1c measurements	4 [4–5]	4 [4–5]	5 [4–5]	*Z* = 2.120	0.034
Exactly four HbA1c measurements, *n* (%)	194 (67.8)	146 (69.9)	48 (62.3)	*χ²* = 1.458	0.227
Span between the first and last measurements, days	330.8 ± 20.5	330.0 ± 21.0	333.0 ± 19.0	*t* = 1.099	0.273
HbA1c standard deviation (HbA1c-SD), percentage points	0.53 [0.40–0.69]	0.49 [0.37–0.64]	0.64 [0.51–0.84]	*Z* = 5.280	<0.001
HbA1c coefficient of variation (HbA1c-CV), %	7.0 [5.4–9.3]	6.5 [5.0–8.4]	8.2 [6.7–10.7]	*Z* = 5.640	<0.001
Variability independent of the mean (VIM)	11.8 [8.9–15.4]	11.0 [8.3–14.4]	14.1 [11.2–18.5]	*Z* = 5.200	<0.001
Residual standard deviation, percentage points	0.40 [0.29–0.56]	0.37 [0.27–0.52]	0.49 [0.36–0.67]	*Z* = 4.520	<0.001
Insulin, *n* (%)	129 (45.1)	82 (39.2)	47 (61.0)	*χ²* = 10.805	0.001
Sulfonylureas or glinides, *n* (%)	88 (30.8)	57 (27.3)	31 (40.3)	*χ²* = 4.455	0.035
Treatment carrying a high risk of hypoglycemia, *n* (%)	171 (59.8)	108 (51.7)	63 (81.8)	*χ²* = 21.267	<0.001
Sedative–hypnotics, *n* (%)	46 (16.1)	26 (12.4)	20 (26.0)	*χ²* = 7.636	0.006
Antidepressants, *n* (%)	27 (9.4)	16 (7.7)	11 (14.3)	*χ²* = 2.893	0.089
Antipsychotics, *n* (%)	9 (3.1)	4 (1.9)	5 (6.5)	Fisher’s exact test†	0.062
Opioids, *n* (%)	13 (4.5)	7 (3.3)	6 (7.8)	Fisher’s exact test†	0.119
Antihypertensives, *n* (%)	221 (77.3)	158 (75.6)	63 (81.8)	*χ²* = 1.240	0.266
Diuretics, *n* (%)	58 (20.3)	35 (16.7)	23 (29.9)	*χ²* = 5.995	0.014
Total number of long-term medications (*n* = 283; 207/76)	6 [4–8]	6 [4–8]	7 [5–9]	*Z* = 3.110	0.002
Number of fall-risk-increasing medication classes	1 [1–2]	1 [1–2]	2 [1–3]	*Z* = 3.750	<0.001
Number of changes to the glucose-lowering regimen (*n* = 283; 207/76)	1 [0–2]	1 [0–2]	2 [1–3]	*Z* = 3.960	<0.001

*N*, total sample size; *n*, number of patients in a group; HbA1c, glycated hemoglobin A1c; SD, standard deviation; CV, coefficient of variation; HbA1c-SD, glycated hemoglobin A1c standard deviation; HbA1c-CV, glycated hemoglobin A1c coefficient of variation; VIM, variability independent of the mean; IQR, interquartile range; *t*, independent-samples *t*-test statistic; *χ²*, Pearson chi-square statistic; *Z*, standardized statistic for the Mann–Whitney *U* test; *β*, exponent obtained by regressing ln(SD) on ln(mean HbA1c), with a value of 0.74. †Two-sided Fisher’s exact tests were used because of small expected cell counts in the contingency tables; these tests do not yield a conventional *χ²* statistic. The values 207/76 in parentheses indicate the numbers of patients with available data in the groups without/with recurrent falls, respectively. Continuous variables are presented as mean ± SD or median [IQR].

### 3.3 Fall outcomes and 365-day risk

During the observation period, 127 patients experienced at least one fall, 77 experienced recurrent falls, and 240 falls were recorded in total. Across increasing HbA1c-CV tertiles, the proportion of patients with no falls decreased, whereas the proportion with one fall did not change monotonically. The distributions of two, three, four, and five falls generally shifted toward the higher tertiles. The proportion with any fall, the risk of recurrent falls, the total number of falls, and the fall incidence rate all increased across tertiles (any fall, *P* = 0.011; recurrent falls, *P* < 0.001; fall incidence rate, *P* < 0.001) (**Table 3**).

**Table 3:** Fall outcomes across tertiles of the glycated hemoglobin A1c coefficient of variation (HbA1c-CV).

Outcome	Lowest tertile (T1) (*n* = 95)	Middle tertile (T2) (*n* = 95)	Highest tertile (T3) (*n* = 96)	Overall (*N* = 286)	Statistic and *P* value
0 falls, *n* (%)	63 (66.3)	53 (55.8)	43 (44.8)	159 (55.6)	—
1 fall, *n* (%)	17 (17.9)	18 (18.9)	15 (15.6)	50 (17.5)	—
2 falls, *n* (%)	12 (12.6)	16 (16.8)	22 (22.9)	50 (17.5)	—
3 falls, *n* (%)	3 (3.2)	7 (7.4)	10 (10.4)	20 (7.0)	—
4 falls, *n* (%)	0 (0.0)	0 (0.0)	5 (5.2)	5 (1.7)	—
5 falls, *n* (%)	0 (0.0)	1 (1.1)	1 (1.0)	2 (0.7)	—
Any fall, *n* (%)	32 (33.7)	42 (44.2)	53 (55.2)	127 (44.4)	*χ²* = 8.963; *P* = 0.011
Recurrent falls, *n* (%)	15 (15.8)	24 (25.3)	38 (39.6)	77 (26.9)	*χ²* = 13.939; *df* = 2; *P* < 0.001
365-day risk, % (95% confidence interval [CI])	15.8 (9.8–24.4)	25.3 (17.6–34.8)	39.6 (30.4–49.6)	26.9 (22.1–32.3)	Pearson *χ²* = 13.939; *df* = 2; *P* < 0.001
Total number of falls	50	76	114	240	—
Observation time, person-years	95.0	95.0	96.0	286.0	—
Fall incidence rate, falls/100 person-years	52.6	80.0	118.8	83.9	Wald *χ²* = 15.752; *df* = 2; *P* < 0.001

HbA1c, glycated hemoglobin A1c; *N*, total sample size; *n*, number of patients in a group; HbA1c-CV, glycated hemoglobin A1c coefficient of variation; T1, T2, and T3, the lowest, middle, and highest HbA1c-CV tertiles, respectively; CI, confidence interval; *df*, degrees of freedom; *χ²*, chi-square statistic; Wald *χ²*, Wald chi-square statistic for the overall effect in negative binomial regression. The 365-day risk was calculated as the number of patients with recurrent falls divided by the number of patients in each tertile, with 95% CIs calculated using the Wilson method. Between-group comparisons of any fall and recurrent falls used Pearson’s *χ²* tests. The between-group test of fall incidence rates was obtained from unadjusted negative binomial regression. Observation time is expressed in person-years.

The 365-day risks of recurrent falls in the lowest (T1), middle (T2), and highest (T3) tertiles were 15.8% (95% CI, 9.8%–24.4%), 25.3% (95% CI, 17.6%–34.8%), and 39.6% (95% CI, 30.4%–49.6%), respectively. The between-group difference was statistically significant (Pearson *χ²* = 13.939; degrees of freedom [*df*] = 2; *P* < 0.001) (**Figure 2**).

**Figure 2:**
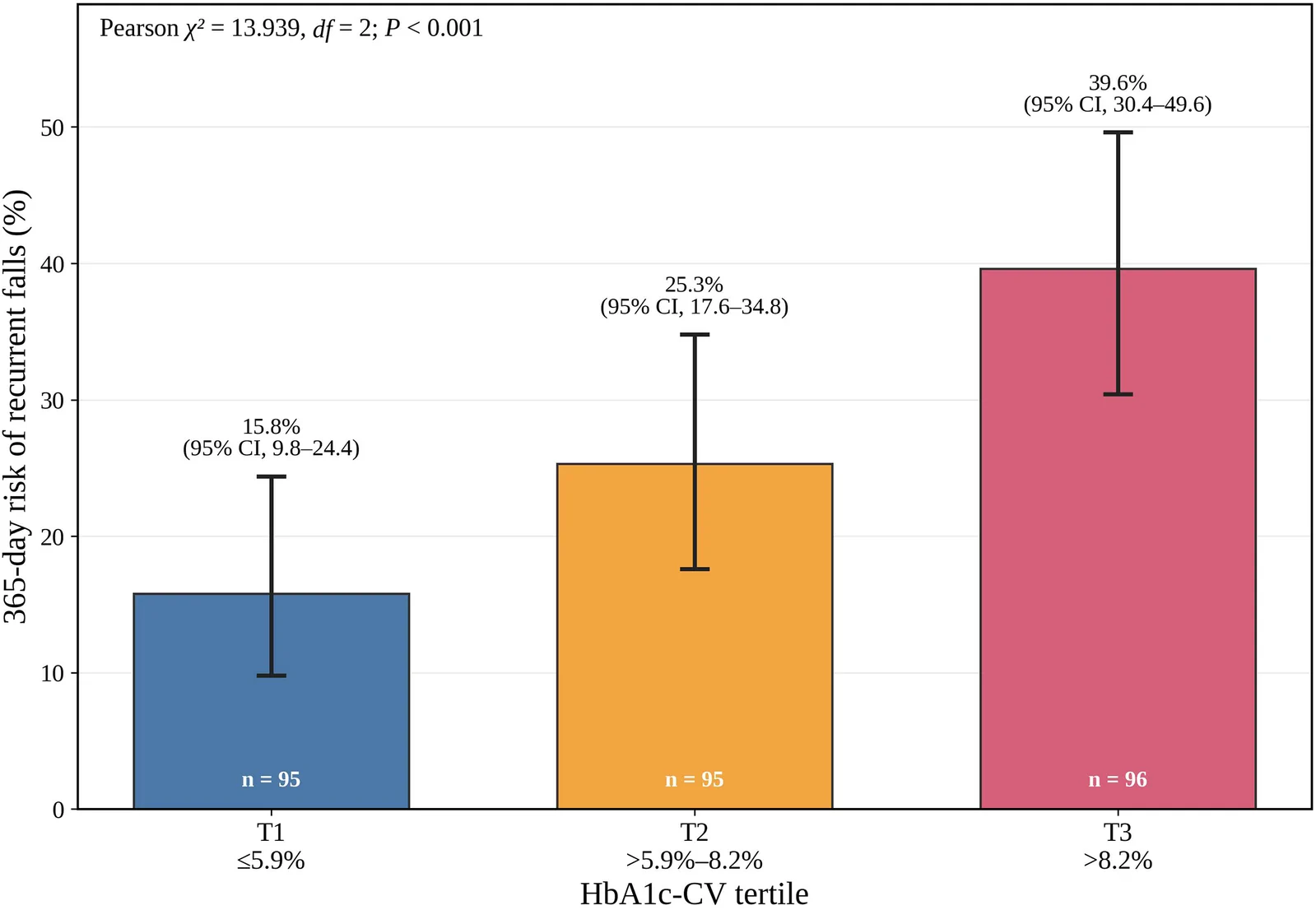
The 365-day risk of recurrent falls across tertiles of the glycated hemoglobin A1c coefficient of variation (HbA1c-CV). Bar heights indicate the 365-day risk of recurrent falls, and error bars indicate Wilson 95% confidence intervals (CIs). The lowest (T1), middle (T2), and highest (T3) tertiles correspond to HbA1c-CV ≤5.9%, >5.9% to ≤8.2%, and >8.2%, respectively. Between-group comparisons used Pearson’s *χ²* test. HbA1c, glycated hemoglobin A1c; *χ²*, chi-square statistic; *df*, degrees of freedom; *n*, number of patients.

### 3.4 Primary association between HbA1c-CV and recurrent falls

In the unadjusted analysis, each 1-SD increase in HbA1c-CV was associated with a higher risk of recurrent falls (RR, 1.48; 95% CI, 1.30–1.69; *P* < 0.001). Compared with T1, the risk for T2 did not differ significantly (RR, 1.60; 95% CI, 0.90–2.85; *P* = 0.112), whereas T3 had a higher risk (RR, 2.51; 95% CI, 1.48–4.24; *P* = 0.001). The overall effect of the tertile categorical variable was statistically significant (Wald *χ²* = 13.118; *df* = 2; *P* = 0.001).

After adjustment for the prespecified confounders, each 1-SD increase in HbA1c-CV was associated with a 34% higher risk of recurrent falls (RR, 1.34; 95% CI, 1.16–1.55; *P* < 0.001). The difference between T2 and T1 was not statistically significant (RR, 1.34; 95% CI, 0.73–2.48; *P* = 0.348), whereas the risk was higher in T3 (RR, 2.03; 95% CI, 1.19–3.47; *P* = 0.010). The overall effect of the tertile categorical variable was statistically significant (*P* = 0.018) (**Table 4**; **Figure 3**).

**Table 4:** Modified Poisson regression analysis of the association between the glycated hemoglobin A1c coefficient of variation (HbA1c-CV) and recurrent falls.

Exposure specification	Model	Risk ratio (RR) (95% confidence interval [CI])	Wald statistic	*P* value	Notes
Per one sample standard deviation (SD)	Unadjusted	1.48 (1.30–1.69)	*Z* = 5.858	<0.001	1 SD = 3.1 percentage points
Per 1-SD increase	Adjusted	1.34 (1.16–1.55)	*Z* = 3.958	<0.001	Primary effect
Lowest tertile (T1)	Unadjusted	1.00 (reference)	—	—	Reference group
Middle tertile (T2)	Unadjusted	1.60 (0.90–2.85)	*Z* = 1.591	0.112	—
Highest tertile (T3)	Unadjusted	2.51 (1.48–4.24)	*Z* = 3.424	0.001	—
Overall tertile effect	Unadjusted	—	Wald *χ²* = 13.118, *df* = 2	0.001	—
T1	Adjusted	1.00 (reference)	—	—	Reference group
T2	Adjusted	1.34 (0.73–2.48)	*Z* = 0.938	0.348	—
T3	Adjusted	2.03 (1.19–3.47)	*Z* = 2.593	0.010	—
Overall tertile effect	Adjusted	—	Wald *χ²* = 7.980, *df* = 2	0.018	—

HbA1c, glycated hemoglobin A1c; HbA1c-CV, glycated hemoglobin A1c coefficient of variation; RR, risk ratio; CI, confidence interval; SD, standard deviation; T1, T2, and T3, the lowest, middle, and highest HbA1c-CV tertiles, respectively; Wald *Z*, Wald standard normal statistic for an individual regression coefficient; Wald *χ²*, Wald chi-square statistic for the overall effect of a categorical variable; *df*, degrees of freedom. RRs were estimated using modified Poisson regression with a log link and robust sandwich variance. The adjusted model included age, mean HbA1c, the number of HbA1c measurements, the span between the first and last measurements, a history of falls during the exposure assessment window, a history of severe hypoglycemia, limitations in activities of daily living, and treatment carrying a high risk of hypoglycemia.

**Figure 3:**
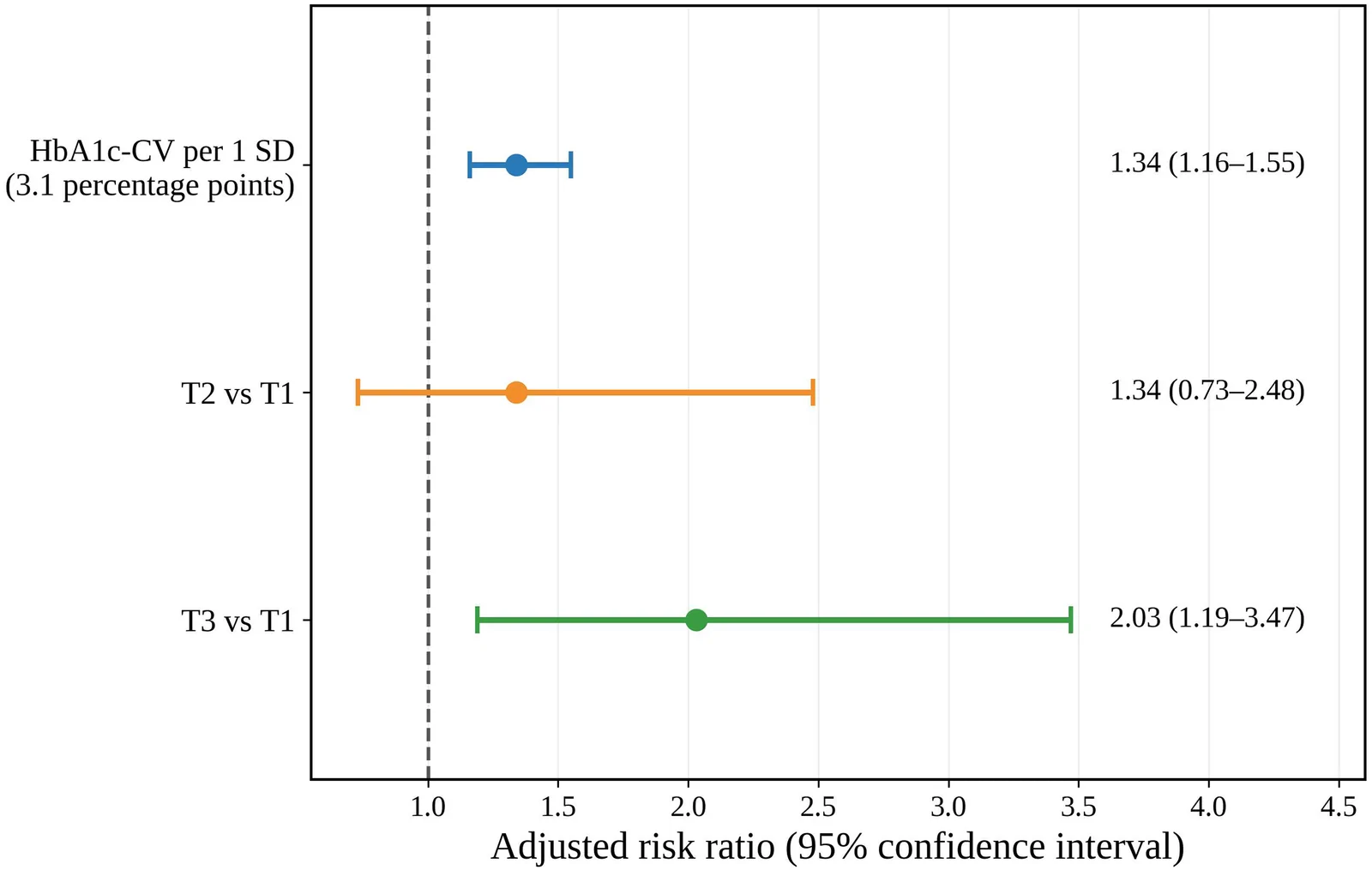
Adjusted association between the glycated hemoglobin A1c coefficient of variation (HbA1c-CV) and recurrent falls. Points indicate adjusted risk ratios (RRs), and horizontal lines indicate 95% confidence intervals (CIs). HbA1c-CV was analyzed per increase of one sample standard deviation (SD; 3.1 percentage points) and by tertile, with the lowest tertile (T1) as the reference. T2 and T3 represent the middle and highest tertiles, respectively. The adjustment variables were the same as those listed in **Table 4**. HbA1c, glycated hemoglobin A1c.

Restricted cubic spline analysis showed a statistically significant overall association (*χ²* = 13.280; *df* = 2; *P* = 0.001), but the test for nonlinearity was not statistically significant (*χ²* = 1.100; *df* = 1; *P* = 0.294), providing no clear evidence of a nonlinear association (**Figure 4**).

**Figure 4:**
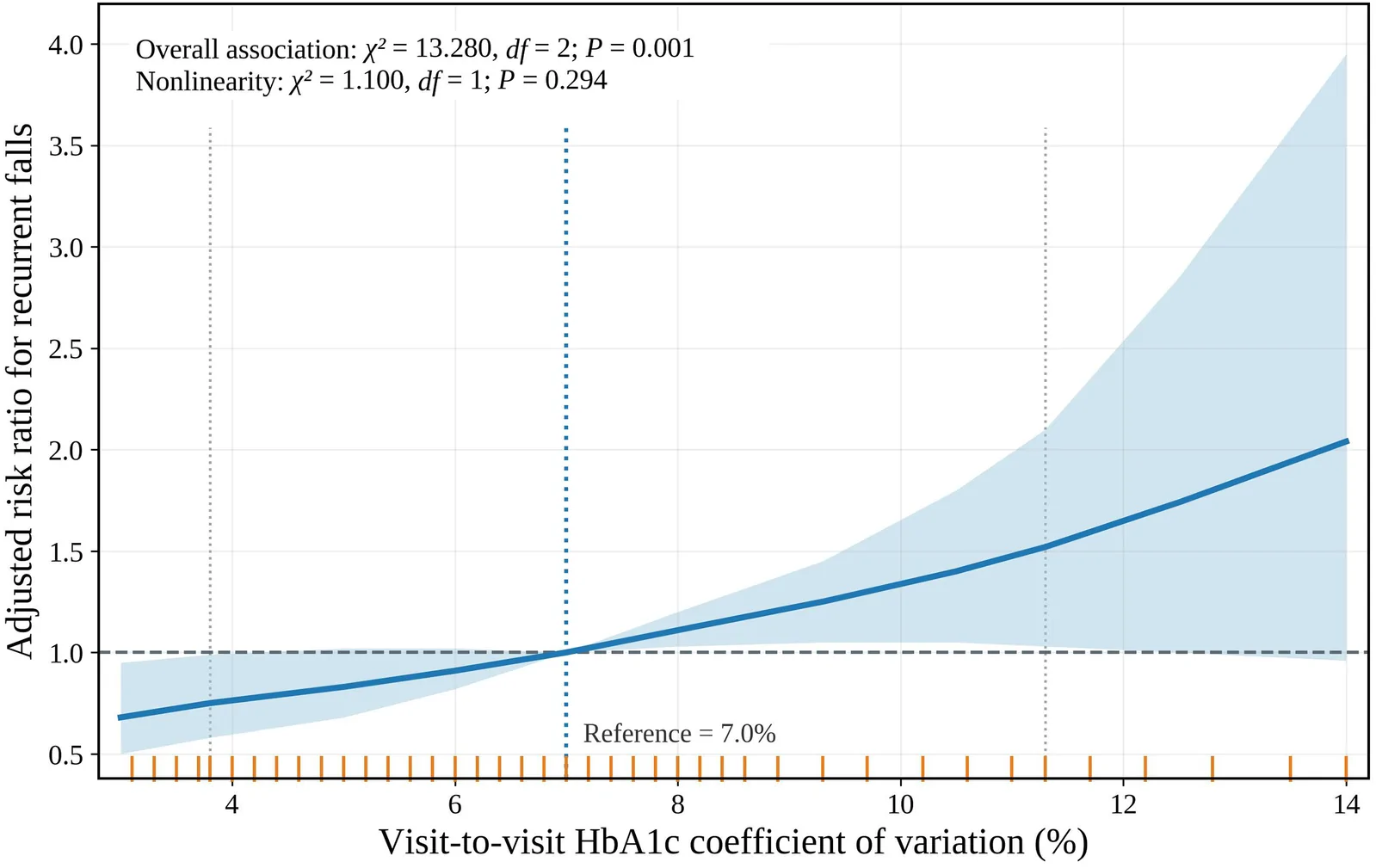
Restricted cubic spline association between the glycated hemoglobin A1c coefficient of variation (HbA1c-CV) and the risk of recurrent falls. The solid line indicates the adjusted risk ratio (RR), the shaded area indicates the pointwise 95% confidence interval (CI), and the short vertical marks on the horizontal axis indicate the sample distribution of HbA1c-CV. Knots were placed at 3.8%, 7.0%, and 11.3%, with 7.0% as the reference. The adjustment variables were the same as those listed in **Table 4**. *P* = 0.001 for the overall association and *P* = 0.294 for nonlinearity. HbA1c, glycated hemoglobin A1c; *χ²*, chi-square statistic; *df*, degrees of freedom.

### 3.5 Fall count analysis

In the adjusted negative binomial regression analysis, each 1-SD increase in HbA1c-CV was associated with a higher fall incidence rate (IRR, 1.37; 95% CI, 1.18–1.58; *P* < 0.001). Compared with T1, the difference for T2 was not statistically significant (IRR, 1.31; 95% CI, 0.90–1.90; *P* = 0.157), whereas T3 had a higher fall incidence rate (IRR, 1.91; 95% CI, 1.32–2.77; *P* = 0.001). The overall effect of the tertile categorical variable was statistically significant (*P* = 0.002). The dispersion parameter *α* of the unadjusted negative binomial model was 0.67 (95% CI, 0.27–1.08), and the Pearson dispersion statistic of the corresponding Poisson model was 1.41. The negative binomial model provided a better fit than the Poisson model (* $\bar{\text{χ}}$²* = 21.513; *P* < 0.001) (**Table 5**).

**Table 5:** Negative binomial regression analysis of the association between the glycated hemoglobin A1c coefficient of variation (HbA1c-CV) and fall counts.

Exposure specification	Incidence rate ratio (IRR) (95% confidence interval [CI])	Test statistic	*P* value	Model diagnostics	Result
Per one sample standard deviation (SD)	1.37 (1.18–1.58)	*Z* = 4.228	<0.001	—	Higher fall incidence rate
Lowest tertile (T1)	1.00 (reference)	—	—	—	Reference group
Middle tertile (T2)	1.31 (0.90–1.90)	*Z* = 1.417	0.157	—	No statistically significant difference
Highest tertile (T3)	1.91 (1.32–2.77)	*Z* = 3.422	0.001	—	Higher fall incidence rate
Overall tertile effect	—	Wald *χ²* = 12.400, *df* = 2	0.002	—	Statistically significant overall effect
Overdispersion assessment	—	* $\bar{\text{χ}}$²* = 21.513	<0.001	*α* = 0.67 (0.27–1.08); Pearson = 1.41	Better fit of the negative binomial model

HbA1c, glycated hemoglobin A1c; HbA1c-CV, glycated hemoglobin A1c coefficient of variation; IRR, incidence rate ratio; CI, confidence interval; SD, standard deviation; T1, T2, and T3, the lowest, middle, and highest HbA1c-CV tertiles, respectively; Wald *Z*, Wald standard normal statistic for an individual regression coefficient; Wald *χ²*, Wald chi-square statistic for the overall effect of a categorical variable; *df*, degrees of freedom; *α*, dispersion parameter of the negative binomial model; Pearson, the ratio of the Pearson *χ²* statistic to the residual degrees of freedom in the Poisson model; *$\bar{\text{χ}}$²*, the likelihood ratio statistic evaluated against a mixture chi-square distribution because *α* = 0 lies on the boundary of the parameter space. All IRRs were obtained from adjusted negative binomial regression, with the same adjustment variables as in **Table 4**. Overdispersion diagnostics were obtained from unadjusted models containing only HbA1c-CV tertiles. The parameter *α* was estimated from the negative binomial model, and the Pearson dispersion statistic was calculated from the corresponding Poisson model.

### 3.6 Sensitivity analyses, missing data, and model diagnostics

The direction of the association remained consistent with the primary analysis when HbA1c-CV was replaced by HbA1c-SD, VIM, or residual SD; when the analysis was restricted to patients with exactly four HbA1c measurements; when patients who had fallen during the exposure assessment window were excluded; in the complete-case analysis; and in the ridge-penalized model with an expanded set of adjustment variables (**Table 6**).

**Table 6:** Sensitivity analyses of the association between glycated hemoglobin A1c (HbA1c) variability and recurrent falls.

Analysis	*n*	Recurrent falls	Risk ratio (RR) (95% confidence interval [CI])	Statistic	*P* value
Primary analysis: per 1-standard-deviation (SD) increase in the HbA1c coefficient of variation (HbA1c-CV)	286	77	1.34 (1.16–1.55)	*Z* = 3.958	<0.001
Per 1-SD increase in HbA1c standard deviation (HbA1c-SD)	286	77	1.30 (1.13–1.50)	*Z* = 3.631	<0.001
Per 1-SD increase in variability independent of the mean (VIM)	286	77	1.32 (1.14–1.53)	*Z* = 3.699	<0.001
Per 1-SD increase in residual SD	286	77	1.28 (1.11–1.48)	*Z* = 3.364	0.001
Exactly four HbA1c measurements	194	48	1.31 (1.06–1.62)	*Z* = 2.496	0.013
Excluding patients with falls during the exposure assessment window	198	34	1.29 (1.01–1.65)	*Z* = 2.034	0.042
Complete-case analysis of the primary model	282	76	1.35 (1.17–1.57)	*Z* = 4.000	<0.001
Ridge-penalized model with an expanded adjustment set	269	73	1.32 (1.12–1.55)	—	—

HbA1c, glycated hemoglobin A1c; *n*, number of patients included in the analysis; HbA1c-CV, glycated hemoglobin A1c coefficient of variation; HbA1c-SD, glycated hemoglobin A1c standard deviation; VIM, variability independent of the mean; RR, risk ratio; CI, confidence interval; SD, standard deviation; *Z*, Wald standard normal statistic. Each alternative exposure measure was standardized using its own sample SD. The model restricted to patients with exactly four measurements did not include the number of HbA1c measurements; the model excluding patients who had fallen during the exposure assessment window did not include previous falls. Except for the ridge-penalized model, RRs were estimated using modified Poisson regression with robust sandwich variance. Bootstrap 95% CIs were used for the ridge-penalized model, and conventional *P* values were not reported.

Body mass index was missing for seven patients, eGFR for six, activities-of-daily-living status for four, and the total number of long-term medications and the number of changes to the glucose-lowering regimen for three each. Seventeen patients had at least one missing variable, including five with two or more missing variables. There were no missing data for the primary exposure or outcomes. Among the variables included in the primary model, only activities-of-daily-living status had missing values; therefore, 282 patients were included in the complete-case analysis. The overall range of variance inflation factors across the 20 imputed datasets was 1.06–2.18, indicating no substantial multicollinearity.

## 4 Discussion

This study showed that visit-to-visit HbA1c-CV remained associated with recurrent falls over the subsequent 12 months after adjustment for mean HbA1c and multiple fall-related factors. Each 1-SD increase in HbA1c-CV was associated with a 34% higher risk of recurrent falls, and the adjusted RR for the highest versus the lowest tertile was 2.03. Restricted cubic spline analysis showed a statistically significant overall association without clear evidence of nonlinearity. The direction of the association was consistent in the fall count analysis and sensitivity analyses. Fall risk in older adults with type 2 diabetes is influenced jointly by age, disease duration, functional limitations, and treatment-related factors; the present findings are consistent with this multifactorial risk framework (20).

Mean HbA1c reflects overall glycemic exposure, whereas visit-to-visit HbA1c variability reflects longitudinal stability; these measures may therefore provide different dimensions of clinical information. Long-term HbA1c fluctuations are associated with several macrovascular and microvascular complications, supporting the value of glycemic stability as a risk indicator beyond the mean level (21). Repeated transitions between high and low glucose levels may promote oxidative stress, endothelial dysfunction, and microcirculatory injury, thereby affecting skeletal muscle perfusion, nerve conduction, and postural regulation (22). An independent association persisted after adjustment for mean HbA1c in this study, and analyses using HbA1c-SD, VIM, and residual SD yielded associations in the same direction, suggesting that the findings were not dependent on a single variability measure.

Hypoglycemia can directly affect consciousness, attention, and protective responses and is an important component of fall risk in older adults with diabetes. Among older outpatients with type 2 diabetes, hypoglycemia is closely associated with treatment regimens, comorbidities, and functional status (23). Greater long-term variability in HbA1c and fasting glucose is associated with an increased risk of hypoglycemia requiring assistance from another person or medical treatment (24). Although this study adjusted for a history of severe hypoglycemia and treatment carrying a high risk of hypoglycemia, HbA1c-CV may still reflect treatment intensity, meal regularity, medication adherence, and short-term glycemic fluctuations not captured in routine records.

Loss of protective plantar sensation due to diabetic peripheral neuropathy is associated with an increased risk of falls and fractures (25). In an HbA1c-based metabolic stratification study, peripheral neuropathy and the overall complication burden were more prevalent in groups with higher HbA1c levels (26). These cross-sectional findings support an association between glycemic status and neuropathy but do not establish causality or directly address visit-to-visit HbA1c variability or falls. Long-term HbA1c fluctuations may further reduce neuromuscular reserve through recurrent metabolic and oxidative stress. The higher prevalence of diabetic peripheral neuropathy among patients with recurrent falls in this study is compatible with this potential pathway but does not demonstrate mediation. The association between HbA1c variability and dementia risk suggests that impairments in executive function, allocation of attention, and hazard recognition may also contribute to falls (27). Visual impairment is likewise associated with increased fall risk in older adults (28), consistent with the higher prevalence of visual impairment among patients with recurrent falls in this study. These baseline characteristics describe the clinical risk profile; their specific mediating roles require evaluation in dedicated analyses.

Declining kidney function may be accompanied by reduced muscle strength, shorter step length, and altered drug clearance, and shorter step length is associated with fall risk in patients with chronic kidney disease (29). Chronic kidney disease and polypharmacy are independently associated with recurrent falls (30). In this study, patients with recurrent falls had lower eGFR, used more long-term medications, and more frequently used sedative–hypnotics and diuretics, further emphasizing that fall-risk assessment should encompass organ function and medication burden. Falls in older adults with type 2 diabetes are also associated with previous falls, cognition, depression, functional status, and walking ability (31). In the present study, previous falls, limitations in activities of daily living, walking-aid use, and cognitive impairment all showed between-group differences in the same direction.

Compared with a single fall, recurrent falls more closely reflect persistent functional and psychological burden and are associated with increased subsequent healthcare utilization (32). Orthostatic hypotension may present as symptoms on standing, cognitive slowing, or unexplained falls, and standardized supine and standing blood pressure measurements can help identify this risk (33). Medication review and deprescribing are appropriate components of multifactorial fall prevention and should be combined with interventions targeting function, balance, and the environment (34). Frailty is closely associated with fall risk in older adults with type 2 diabetes (35), suggesting that the clinical interpretation of HbA1c-CV should be situated within comprehensive geriatric assessment. Deficits in peripheral nerve axonal transport, myelin maintenance, and mitochondrial function may contribute to the progression of diabetic peripheral neuropathy (36), providing further biological support for the links among abnormal HbA1c, declining neuromuscular function, and falls.

Clinically, HbA1c-CV may provide complementary information for multidimensional fall-risk assessment. In patients with persistently elevated HbA1c-CV, previous falls, hypoglycemic exposure, neuropathy, vision, cognition, blood pressure during postural changes, kidney function, medication burden, gait, and balance may be reviewed concurrently, and treatment regimens may be optimized in conjunction with individualized glycemic targets.

### 4.1 Study limitations

This study has several limitations. First, retrospective data cannot fully account for residual confounding. Information on diet, physical activity, sarcopenia, vestibular function, and the home environment was incomplete, precluding causal inference. Second, HbA1c measurements were obtained during routine care, and their number and timing were not fully standardized. Although the models adjusted for the number of measurements and the span between the first and last measurements, and consistent results were obtained among patients with exactly four measurements, measurement-frequency-related bias may remain. Third, falls were ascertained from existing medical and follow-up records; events that did not cause injury or were not spontaneously reported may have been missed. Fourth, the primary model included several prespecified covariates, whereas the number of recurrent-fall events was limited, resulting in wide confidence intervals for some estimates. Finally, inclusion required at least four eligible HbA1c measurements and verifiable fall data. Generalization to other healthcare settings, age groups, or long-term care populations therefore requires consideration of population characteristics.

## 5 Conclusion

Greater visit-to-visit HbA1c variability in older adults with type 2 diabetes was associated with recurrent falls and a higher number of falls over 12 months. This association persisted after adjustment for mean HbA1c, previous falls, severe hypoglycemia, and treatment carrying a high risk of hypoglycemia. HbA1c-CV may provide complementary information for routine fall-risk assessment. Its clinical utility and the effects of reducing variability on fall outcomes warrant further investigation.

## Data Availability

The data supporting the findings of this study are available from the corresponding author upon reasonable request.
